# Higher Risk of Thyroid Disorders in Young Patients with Type 1 Diabetes: A 12-Year Nationwide, Population-Based, Retrospective Cohort Study

**DOI:** 10.1371/journal.pone.0152168

**Published:** 2016-03-23

**Authors:** Ming-Chi Lu, Shou-Chih Chang, Kuang-Yung Huang, Malcolm Koo, Ning-Sheng Lai

**Affiliations:** 1 Division of Allergy, Immunology and Rheumatology, Dalin Tzu Chi Hospital, Buddhist Tzu Chi Medical Foundation, Dalin, Chiayi, 62247, Taiwan; 2 School of Medicine, Tzu Chi University, Hualien, 97004, Taiwan; 3 Division of Pediatrics, Dalin Tzu Chi Hospital, Buddhist Tzu Chi Medical Foundation, Dalin, Chiayi, 62247, Taiwan; 4 Department of Medical Research, Dalin Tzu Chi Hospital, Buddhist Tzu Chi Medical Foundation, Dalin, Chiayi, 62247, Taiwan; 5 Dalla Lana School of Public Health, University of Toronto, Toronto, Ontario, M5S 3G3, Canada; Universidade Federal do Rio de Janeiro, BRAZIL

## Abstract

**Background:**

The association between type 1 diabetes and thyroid autoimmunity has been studied in various populations, but seldom on Taiwanese children and adolescents. Therefore, the aim of this study was to examine the incidence of autoimmune thyroid disorders in Taiwanese children and adolescent patients with type 1 diabetes, based on data from a nationwide, population-based, health claims database.

**Methods:**

Using Taiwan’s National Health Insurance Research Database, we identified 3,652 patients with type 1 diabetes between 2000 and 2012. A comparison cohort was assembled, which consisted of five patients without type 1 diabetes, based on frequency matching for sex and 3-year age interval, for each patient with type 1 diabetes. Both groups were followed until diagnosis of thyroid disorders or the end of the follow-up period. Poisson regression models were used to calculate incidence rate ratios for the thyroid disorders between the type 1 diabetes cohort and the comparison cohort.

**Results:**

Simple and unspecified goiter (International Classification of Diseases, 9^th^ Revision, Clinical Modification [ICD-9-CM] code 240), thyrotoxicosis (ICD-9-CM code 242), unspecified hypothyroidism (ICD-9-CM code 244.9), and thyroiditis (ICD-9-CM code 245) showed significantly higher incidences in the type 1 diabetes cohort compared with the control cohort, with incidence rate ratios of 2.74, 6.95, 6.54, 16.07, respectively.

**Conclusions:**

Findings from this nationwide, population-based cohort study showed that the incidences of autoimmune thyroid disorders were significantly higher in Taiwanese children and adolescents with type 1 diabetes compared with those without the disease.

## Introduction

Type 1 diabetes, an autoimmune disease, is the most common type of diabetes in children and adolescents. It is also the most common chronic disease in children in the developed countries [1]. The illness is characterized by the body’s inability to produce insulin due to the autoimmune destruction of the beta cells in the pancreas. Globally, it is estimated that there are almost 500,000 children aged under 15 years with type 1 diabetes, with large geographical variations in incidence [2]. In Taiwan, its bi-annual incidence per 100,000 population in 2009–2010 among those under the age of 15 years was reported to be 5.88 and 6.92 for male and female patients, respectively [3].

Type 1 diabetes is associated with microvascular complications, including diabetic neuropathy, and diabetic retinopathy as well as macrovascular diseases, such as cardiovascular disease and peripheral artery disease [4]. In children, autoimmune thyroid disorders are the most prevalent endocrinopathy among patients with type 1 diabetes. The similar pathogenesis of the two disorders and their frequent clustering within families and individuals, suggest that they may have a shared genetic etiology [5]. Human leukocyte antigen (HLA) class II, cytotoxic T-lymphocyte antigen 4 (CTLA-4), and protein tyrosine phosphatase non-receptor type 22 (PTPN22) have been suggested as potential genetic susceptibility loci [6].

A study on 233 Brazilian children and adolescents with type 1 diabetes found that 23% of them had autoimmune thyroid disorders, with the majority being female and older than 5 years of age [7]. Another study on 382 Polish children and adolescents with type 1 diabetes reported 14.4% of the patients had elevated concentrations of antibodies against thyroid peroxidase [8]. A nationwide cohort study of children and adolescents with type 1 diabetes, treated in pediatric diabetes centers in Germany and Austria, showed that thyroid antibody levels were elevated in 1,530 of the 7,097 patients (22%). Of the patients with positive antibodies, 63% were females, whereas only 45% were females among those without antibodies (*P* < 0.001) [9]. In addition, the prevalence of thyroid antibody titers increased with age [8]. Moreover, a study on 491 children recruited from the Barbara Davis Center for Childhood Diabetes in Colorado found that 122 (24.8%) were positive for thyroid peroxidase autoantibodies [10]. A study on 115 Korean adolescent patients with type 1 diabetes showed a 25% prevalence of autoimmune thyroid disorders in patients with type 1 diabetes, compared with 8% in their age and sex-matched normal controls [11].

Although the association between type 1 diabetes and the occurrence of thyroid autoantibodies has well been established in various populations, relatively few studies have been conducted in Taiwanese children and adolescents [12–14]. In addition, none have assessed the incidence rate of thyroid disorders among Taiwanese children and adolescents with type 1 diabetes and compared with that of the general population. Therefore, the aim of the present work was to examine the incidence rate of thyroid disorders in young Taiwanese patients with type 1 diabetes, based on data from a nationwide, population-based health claims database.

## Methods

### Study design and data sources

We used population-based, retrospective cohort study design to conduct a secondary data analysis based on the data available from the Taiwan's National Health Insurance Research Database (NHIRD) [15]. Two NHIRD datafiles, the 2000 Longitudinal Health Insurance Research Database (LHID 2000) and the catastrophic illness datafile were used. The LHID 2000 contained health claims data for one million beneficiaries randomly sampled from all health insurance enrollees in the Registry of Beneficiaries of the NHIRD in 2000. In Taiwan, a number of serious health conditions, including type 1 diabetes, are considered as catastrophic illnesses by the Ministry of Health and Welfare. Patients with these illnesses can apply for a catastrophic illness certificate. When approved, certificate holders are exempted from insurance premiums and catastrophic illness-related co-payments of health care costs.

The study protocol was reviewed and approved by the institutional review board of the Dalin Tzu Chi Hospital, Buddhist Tzu Chi Medical Foundation, Taiwan (No. B10403028). As the NHIRD data files contain only de-identified secondary data, the need for informed consent from individual patient consent was waived by the institutional review board.

### Identification of the type 1 diabetes cohort and a comparison cohort

The catastrophic illness datafile was searched from January 1, 2000 to December 31, 2012 to assemble a type 1 diabetes cohort. Type 1 diabetes was identified based on the International Classification of Diseases, 9^th^ Revision, Clinical Modification (ICD-9-CM) code 250.x1 or 250.x3. Patients older than 18 years at the time of catastrophic illness certificate application were excluded. Patients who had already applied for a type 1 diabetes catastrophic illness certificate between January 1, 1995 to December 31, 1999 were also excluded. In addition, patients who had diagnosed with the thyroid diseases of interest in this study between January 1, 1996 and December 31, 1999 were excluded from the study.

The comparison cohort was assembled by randomly selected patients from the LHID2000 in the year 2000. Five comparison patients were selected, based on frequency matching for sex and 3-year age interval, for each patient with type 1 diabetes. Patients who had diagnosed with the thyroid diseases of interest between January 1, 1996 and December 31, 1999 were excluded from the study.

### Identification of thyroid disorders

Both the type 1 diabetes cohort and the comparison cohort were followed until diagnosis of the outcome thyroid disorders or the end of the follow-up period. The outcome of interest included five thyroid disorders including (1) simple and unspecified goiter (ICD-9-CM code 240), (2) thyrotoxicosis (ICD-9-CM code 242), (3) unspecified hypothyroidism (ICD-9-CM code 244.9), (4) thyroiditis (ICD-9-CM code 245), and (5) non-toxic nodular goiter (ICD-9-CM code 241). In addition, an overall autoimmune thyroid disorder variable were created and the disorder was considered present if the diagnostic code of any of the first four thyroid disorders were present. Non-toxic nodular goiter was not included in the overall autoimmune thyroid disorder variable because it is not an immune disorder by nature. We used a criterion of two diagnoses of the same thyroid disease within a period of 90 days to improve the validity of our study.

### Statistical analysis

Sex and age intervals between the type 1 diabetes cohort and the comparison cohort were compared with Chi-square test. Incidence rate per 100,000 person-years were calculated for each of the five thyroid disorders and the overall autoimmune thyroid disorder separately for the type 1 diabetes cohort and the comparison cohort. Incidence rate ratios (IRR) for the outcome variables were calculated using Poisson regression models (i.e., generalized linear models with a Poisson log-linear link function and person-years as the offset variable). A two-tailed p value of < 0.05 was considered statistically significant. All statistical analyses were conducted using IBM SPSS Statistics software package, version 23.0 (IBM Corp, Armonk, NY, USA).

## Results

We identified 3,652 patients with type 1 diabetes and 18,260 patients without type 1 diabetes for this study and followed up to 12 years post-study entry. Table 1 shows the distribution of sex and age interval at baseline between the type 1 diabetes cohort and the comparison cohort. As the patients in the comparison cohort were frequency matched with those in the type 1 diabetes cohort, there were no significant differences between the two groups.

**Table 1:** Basic characteristics of the type 1 diabetes cohort and comparison cohort (N = 21,912).

Variable	n (%)		*P* value
	Type 1 diabetes cohort	Comparison cohort	
	3,652 (16.7)	18,260 (83.3)	
Sex			> 0.999
male	1,700 (46.5)	8,500 (46.5)	
female	1,952 (53.5)	9,760 (53.5)	
Age at entry (years)			> 0.999
≤ 3.00	216 (5.9)	1,080 (5.9)	
3.01–6.00	472 (12.9)	2,360 (12.9)	
6.01–9.00	551 (15.1)	2,755 (15.1)	
9.01–12.00	831 (22.8)	4,155 (22.8)	
12.01–15.00	885 (24.2)	4,425 (24.2)	
15.01–18.00	697 (19.1)	3,485 (19.1)	

p values were calculated with the Chi-square test.

% are column percentages except in the header row where they are row percentages.

Table 2 displays the incidence rates and IRRs of thyroid disorders for the type 1 diabetes cohort and the comparison cohort. First, the overall autoimmune thyroid disorder (ICD-9-CM codes 240, 242, 2449, and 245) showed a significantly higher incidence in the type 1 diabetes cohort compared with the comparison cohort (IRR of 6.65, p *<* 0.001). Second, the four individual autoimmune thyroid disorders also showed significantly elevated incidence rates in the type 1 diabetes cohort compared to the comparison cohort. The IRRs for simple and unspecified goiter (ICD-9-CM code 240), thyrotoxicosis (ICD-9-CM code 242), unspecified hypothyroidism (ICD-9-CM code 244.9), and thyroiditis (ICD-9-CM code 245) were 2.74, 6.95, 6.54, 16.07, respectively (all p *<* 0.001). Third, the IRR was not significant between the type 1 diabetes and the comparison cohort for nontoxic nodular goiter (ICD-9-CM code 241), a non-autoimmune thyroid disorder.

**Table 2:** Incidence rates and incidence rate ratios of thyroid disorders for type 1 diabetes cohort and comparison cohort.

Isorder (ICD-9-CM)	Type 1 diabetes cohort			Comparison cohort			IRR (95% CI)	p value
	No. of events	Person-years	IR	No. of events	Person-years	IR		
Overall autoimmune thyroid disorder (240, 242, 244.9, 245)	236	22,862	1032.27	361	232,547	155.24	6.65 (5.64–7.84)	< 0.001
Simple and unspecified goiter (240)	45	24,144	186.38	159	233,730	68.03	2.74 (1.97–3.82)	< 0.001
Thyrotoxicosis (242)	142	23,384	607.26	204	233,466	87.38	6.95 (5.61–8.61)	< 0.001
Hypothyroidism, unspecified (244.9)	27	24,204	111.55	40	234,436	17.06	6.54 (4.01–10.65)	< 0.001
Thyroiditis (245)	43	24,133	178.18	26	234,542	11.09	16.07 (9.88–26.16)	< 0.001
Nontoxic nodular goiter (241)	5	24,340	20.54	43	234,448	18.34	1.12 (0.44–2.83)	0.810

IR, incidence rate per 100,000 person-years; IRR, incidence rate ratio, compared type 1 diabetes cohort with comparison cohort; ICD-9-CM, International Classification of Diseases, Ninth Revision, clinical modification.

Table 3 showed the incidence rates and IRRs of autoimmune thyroid disorders for the type 1 diabetes cohort and the comparison cohort, stratified by sex. The IRRs for the overall autoimmune thyroid disorder and the four individual autoimmune thyroid disorders were significant with larger magnitudes in male patients compared with female patients.

**Table 3:** Incidence rates and incidence rate ratios of thyroid disorders for type 1 diabetes cohort and comparison cohort, stratified by sex.

Sex	Disorder (ICD-9-CM)	Type 1 diabetes cohort			Comparison cohort			IRR (95% CI)	p value
		No. of events	Person-years	IR	No. of events	Person-years	IR		
**Male**									
	Overall autoimmune thyroid disorder (240, 242, 244.9, 245)	67	10,862	616.82	61	108,883	56.02	11.01 (7.78–15.58)	< 0.001
	Simple and unspecified goiter (240)	8	11,277	70.94	23	109,100	21.08	3.37 (1.51–7.52)	0.003
	Thyrotoxicosis (242)	48	10,944	438.61	41	109,034	37.60	11.66 (7.69–17.70)	< 0.001
	Hypothyroidism, unspecified (244.9)	3	11,303	26.54	5	109,204	4.58	5.80 (1.39–24.26)	0.016
	Thyroiditis (245)	11	11,271	97.60	3	109,228	2.75	35.54 (9.91–127.38)	< 0.001
**Female**									
	Overall autoimmune thyroid disorder (240, 242, 244.9, 245)	169	12,000	1408.34	300	123,665	242.59	5.81 (4.81–7.01)	< 0.001
	Simple and unspecified goiter (240)	37	12,867	287.57	136	124,631	109.12	2.64 (1.83–3.79)	< 0.001
	Thyrotoxicosis (242)	94	12,440	755.62	163	124,432	131.00	5.77 (4.48–7.44)	< 0.001
	Hypothyroidism, unspecified (244.9)	24	12,902	186.03	35	125,233	27.95	6.66 (3.96–11.19)	< 0.001
	Thyroiditis (245)	32	12,863	248.78	23	125,313	18.36	13.56 (7.93–23.16)	< 0.001

IR, incidence rate per 100,000 person-years; IRR, incidence rate ratio, compared type 1 diabetes cohort with comparison cohort; ICD-9-CM, International Classification of Diseases, Ninth Revision, clinical modification.

The comparison cohort was frequency-matched for 3-year age interval with the type 1 diabetes cohort.

Table 4 showed the incidence rates and IRRs of autoimmune thyroid disorders for the type 1 diabetes cohort and the comparison cohort, stratified by three age intervals. In the 6 to <12 years and 12 to 18 years age intervals, the IRRs for the overall autoimmune thyroid disorder and the four individual autoimmune thyroid disorders were all significant, indicating higher incidence rates in the type 1 diabetes cohort compared with the comparison cohort. In the ≤ 6 years age interval, an IRR for unspecified hypothyroidism was incalculable due to no events in the type 1 diabetes cohort. The IRRs were significant for the overall autoimmune thyroid disorder (p < 0.001) and thyroiditis (p < 0.001) but not significant for simple and unspecified goiter (p = 0.560).

**Table 4:** Incidence rates and incidence rate ratios of thyroid disorders for type 1 diabetes cohort and comparison cohort, stratified by age group.

Age (years)	Disorder (ICD-9-CM)	Type 1 diabetes cohort			Comparison cohort			IRR (95% CI)	p value
		No. of events	Person-years	IR	No. of events	Person-years	IR		
**≤ 6.00**									
	Overall autoimmune thyroid disorder (240, 242, 244.9, 245)	21	4,558	460.71	16	44,245	36.16	12.74 (6.65–24.41)	< 0.001
	Simple and unspecified goiter (240)	1	4,675	21.39	5	44,289	11.29	1.90 (0.22–16.22)	0.560
	Thyrotoxicosis (242)	13	4,597	282.82	11	44,272	24.85	11.38 (5.10–25.41)	< 0.001
	Hypothyroidism, unspecified (244.9)	0	4,686	0	2	44,290	4.52	NA	NA
	Thyroiditis (245)	9	4,641	193.94	1	44,304	2.26	85.92 (10.89–678.21)	< 0.001
**6.01–12.00**									
	Overall autoimmune thyroid disorder (240, 242, 244.9, 245)	78	8,462	921.73	110	88,418	124.41	7.41 (5.54–9.90)	< 0.001
	Simple and unspecified goiter (240)	13	8,910	145.90	47	88,737	52.97	2.76 (1.49–5.09)	0.001
	Thyrotoxicosis (242)	49	8,612	568.98	54	88,693	60.88	9.35 (6.35–13.76)	< 0.001
	Hypothyroidism, unspecified (244.9)	6	8,914	67.31	14	88,881	15.75	4.27 (1.64–11.12)	0.003
	Thyroiditis (245)	16	8,880	180.19	7	88,929	7.87	22.89 (9.42–55.64)	< 0.001
**12.01–18.00**									
	Overall autoimmune thyroid disorder (240, 242, 244.9, 245)	137	9,842	1392.05	235	99,886	235.27	5.92 (4.79–7.30)	< 0.001
	Simple and unspecified goiter (240)	31	10,559	293.60	107	100,705	106.25	2.76 (1.85–4.12)	< 0.001
	Thyrotoxicosis (242)	80	10,175	786.22	139	100,501	138.31	5.69 (4.32–7.48)	< 0.001
	Hypothyroidism, unspecified (244.9)	21	10,604	198.03	24	101,265	23.70	8.36 (4.65–15.01)	< 0.001
	Thyroiditis (245)	18	10,613	169.60	18	101,309	17.77	9.55 (4.97–18.35)	< 0.001

IR, incidence rate per 100,000 person-years; IRR, incidence rate ratio, compared type 1 diabetes cohort with comparison cohort; ICD-9-CM, International Classification of Diseases, Ninth Revision, clinical modification; NA: not available due to no events in the type 1 diabetes cohort.

The comparison cohort was frequency-matched for sex with the type 1 diabetes cohort.

## Discussion

In this retrospective cohort study based on the data from the NHIRD, we examined the incidence of several thyroid disorders among patients with type 1 diabetes over a period of 13 years. Our results support an increased risk of thyroid disorders among children and adolescents with type 1 diabetes in the Taiwanese population. The incidence rate ratio was 6.65 (*P* < 0.001) for the overall autoimmune thyroid disorder, which is a composite variable that represents any of the four following diagnoses: simple and unspecified goiter, thyrotoxicosis, unspecified hypothyroidism, and thyroiditis. The incidence rates were significantly higher for all four autoimmune thyroid disorders in patients with type 1 diabetes compared with those without it. The lack of significant differences in the incidence ratio for nontoxic nodular goiter, which is the enlargement of the thyroid gland not associated with abnormal thyroid function, between the two groups was expected as type 1 diabetes should not increase the risk of non-autoimmune thyroid disorder.

The significant increase in incidence rates for thyrotoxicosis and simple and unspecified goiter is likely to be the results of Graves’ disease [16]. Although ICD-9-CM codes preclude the direct identification of diagnosis of Graves’ disease, it might be deduced based on the following two reasons: First, Graves’ disease accounts for the majority of cases of hyperthyroidism in children and adolescents and hyperthyroidism can lead to thyrotoxicosis. This may also account for the relatively high incidence rate of thyrotoxicosis, regardless of sex and age group, compared to the other thyroid disorders among the patients with type 1 diabetes in our study. Second, Graves’ disease is characterized by diffuse goiter.

The incidence rates of both thyroiditis and unspecified hypothyroidism were found to be significantly higher in patients with type 1 diabetes. Based on our clinical experience, acute or sub-acute thyroiditis was rarely a cause of hypothyroidism. Goiter-related iodine insufficiency was also rare in Taiwan. Therefore, the underlying cause of the observed hypothyroidism was likely to be Hashimoto’s thyroiditis. Moreover, the large IRR of 16.1 for thyroiditis compared with that of thyrotoxicosis is consistent with findings from previous studies that Hashimoto's thyroiditis is a more common clinical presentation compared with Grave's disease in patients with type 1 diabetes [17]. Nevertheless, it should be noted that a high prevalence of iodine-induced non-autoimmune primary hypothyroidism in diabetic patients with advanced diabetic nephropathy, compared with non-diabetic chronic renal dysfunction, had been reported [18].

Our study showed that the IRRs for autoimmune thyroid disorders exhibited larger magnitudes in male patients compared with female patients (Table 3). In the non-type 1 diabetes comparison cohort, the incidence rates of autoimmune thyroid disorders were higher in females compared with males, which confirms findings from previous research that both hyperthyroidism and hypothyroidism disproportionally affect females [19, 20]. In the type 1 diabetes cohort, although the incidence rate was still higher in females, which is consistent with previous research [21], the differences between the sexes were less pronounced. The net result was that larger magnitudes of IRRs were observed among males in all the autoimmune thyroid disorders except unspecified hypothyroidism. The reason for this reversed sex ratio in IRRs is that the incidence rates of the autoimmune thyroid disorders in non-diabetic males are low relative to their female counterparts. Therefore, the increased risk contributed by type 1 diabetes is particularly pronounced in males. Furthermore, since female sex hormone is believed to involve in the pathogenesis of various autoimmune thyroid disorders [22] and since most of our patients with type 1 diabetes were prepubertal, this may explain the observed higher IRR for autoimmune thyroid disorder in males compared to females.

When our study results were present with stratification by age intervals at cohort entry, the IRRs remained significant for the overall autoimmune thyroid disorder and the four individual autoimmune thyroid disorders in the three age intervals with the exception of simple and unspecified goiter and unspecified hypothyroidism in the 6 years or younger age interval. The statistically insignificant results were due to the relatively small number of observations in these two conditions in the 6 years or younger age interval. In addition, the IRRs for the overall autoimmune thyroid disorder, thyrotoxicosis, and thyroiditis showed a pattern of a decrease in magnitude with age. Few reports are available in the literature to compare with our results regarding the age at onset of type 1 diabetes. In a study based on 69 sibling pairs concordant for type 1 diabetes, a younger age at onset of type 1 diabetes was significantly associated with increased occurrence of autoimmune thyroiditis. The prevalence was highest at 36% for those aged 0 to 2 years and progressively declined to 5% for those aged 13 years and above [23]. Nevertheless, a sub-analysis on 66 children in an Italian study reported a higher prevalence of thyroid autoimmunity in those who were pubescent (46%) and postpubertal (41%), compared with those who were prepubertal [24]. As the results from these studies were based on relatively few events, additional investigations are warranted to confirm the findings observed in this study.

Since thyroid hormones are critically related to growth and developmental processes, hypothyroidism and hyperthyroidism are serious conditions in children. Hypothyroidism can cause growth retardation while subclinical hypothyroidism can lead to dislipidemia and cardiovascular complications [25]. On the other hand, hyperthyroidism can worsen glycemic control and increases the risk of diabetic ketoacidosis [26] and neuromuscular dysfunction [27]. Therefore, our findings concur with studies in other populations [7, 28] that screening for thyroid function and autoantibody among patients with type 1 diabetes is recommended. According to the most current practice guidelines, the American Diabetes Association recommends screening children newly diagnosed with type 1 diabetes for autoimmune thyroid disease by measuring antithyroid peroxidase and antithyroglobulin. Thyroid-stimulating hormone concentrations should also be measured. If normal, its levels should be monitored every 1 to 2 years or sooner, particularly if symptoms of thyroid dysfunction, thyromegaly, an abnormal growth rate, or unusual variation in glucose levels are observed [29].

Several limitations should be taken into account when interpreting the results from this study. First, since serological data are not available in the NHIRD, subclinical hypothyroidism or hyperthyroidism cannot be evaluated. Second, no detailed clinical information is available regarding the severity of the autoimmune thyroid disorders and family history of thyroid disorders. Third, the presence of a surveillance bias cannot be ruled out in this study, as patients with type 1 diabetes are more likely to undergo routine blood tests than their non-diabetic counterparts. Despite the above mentioned limitations, the strengths of this study included its nationwide, population-based cohort design, relatively long duration of follow-up, the use of the catastrophic illness database to identify type 1 diabetes, and the large number of incident cases of type 1 diabetes.

In conclusion, this study showed that the incidences of several autoimmune thyroid disorders were significantly higher in children and adolescents with type 1 diabetes compared with those without type 1 diabetes. Type 1 diabetes is the most common endocrine disorder in pediatrics and in view of its increased risk for autoimmune thyroid disorders, it is important for pediatricians to be alert to their subtle signs and symptoms, such as difficulty in maintaining glycemic control, poor growth, palpitations, and heat or cold intolerance, among their patients with type 1 diabetes. Timely detection and management of hypothyroidism and hyperthyroidism are crucial to ensure better diabetes control and improvements in general health can be achieved among patients with type 1 diabetes.
